# Fabrication and Physicochemical Study of B2SA-Grafted Poly(vinyl Alcohol)–Graphene Hybrid Membranes for Dehydration of Bioethanol by Pervaporation

**DOI:** 10.3390/membranes11020110

**Published:** 2021-02-04

**Authors:** Prakash B. Kalahal, Akshay S. Kulkarni, Ashok M. Sajjan, T. M. Yunus Khan, Irfan Anjum Badruddin, Sarfaraz Kamangar, Nagaraj R. Banapurmath, Narasimha H. Ayachit, Manu L. Naik, Vijaykumar S. Marakatti

**Affiliations:** 1 Department of Chemistry, KLE Technological University, Hubballi 580031, India; prakashkalahal@kletech.ac.in (P.B.K.); akshaykulkarni@kletech.ac.in (A.S.K.); manunaik@kletech.ac.in (M.L.N.); 2 Center for Material Science, KLE Technological University, Hubballi 580031, India; nr_banapurmath@kletech.ac.in (N.R.B.); ayachit@kletech.ac.in (N.H.A.); 3 Research Center for Advanced Materials Science (RCAMS), King Khalid University, P.O. Box 9004, Abha 61413, Saudi Arabia; mtatagar@kku.edu.sa (T.M.Y.K.); irfan@kku.edu.sa (I.A.B.); 4 Department of Mechanical Engineering, College of Engineering, King Khalid University, Abha 61421, Saudi Arabia; ssaheb@kku.edu.sa; 5 Institute of Condensed Matter and Nanosciences (IMCN), Molecular Chemistry, Materials and Catalysis (MOST) Université Catholique de Louvain (UCLouvain), 1348 Louvain-la-Neuve, Belgium; vijaykumar.marakatti@uclouvain.be

**Keywords:** poly(vinyl alcohol), B2SA, graphene, bioethanol, pervaporation

## Abstract

Tetraethylorthosilicate (TEOS)-crosslinked poly(vinyl alcohol) (PVA) solution was prepared and treated with benzaldehyde 2 sulphonic sodium salt acid (B2SA) for sulfonation. Different contents of graphene were incorporated into B2SA-grafted PVA–TEOS hybrid membrane to improve the membrane stability, mechanical strength, and overall pervaporation performance of the membranes. Membranes were fabricated using the casting technique. Developed membranes were then analyzed for their physicochemical changes by means of Fourier transform infrared spectroscopy (FTIR), differential scanning calorimetry (DSC), scanning electron microscope (SEM), wide-angle X-ray diffraction (WAXD), thermogravimetric analysis (TGA), contact angle analysis (CA), and mechanical strength. The lower d-spacing value observed in WAXD was evidence for the decreased inter-chain distance between the polymer chains. DSC exhibited the enhanced thermal stability of the developed membranes compared to the plane PVA membrane with enhancement in T*_g_* value (106 °C), which was well above the pervaporation experimental temperature. Incorporation of graphene induced higher mechanical strength to the fabricated membranes. Further, the membranes were tested for the pervaporation separation of bioethanol. All the membranes were stable throughout the pervaporation studies, with M-2 G showing the total permeation flux of 11.66 × 10^−2^ kg/(m^2^ h) at 30 °C.

## 1. Introduction

Bioethanol is one of the attractive renewable fuels that are produced from biomass [1,2,3]. Compared to gasoline, ethanol has higher octane ratings. Hence, bioethanol engines show better thermal efficiency. Top-quality grade ethanol is utilized as an antiseptic and solvent in the medicinal field [4,5]. The azeotropic composition of aqueous bioethanol is challenging to purify with conventional distillation as it is economically not viable, and it makes use of carcinogenic chemicals [6,7]. Instead, the pervaporation, which is a membrane-based process, is preferred because of its efficiency and economic benefits. In pervaporation, membrane plays a significant role in separating the aqueous–organic mixture because of their ability to separate the azeotropic mixture efficiently [8,9].

In pervaporation, the separation efficiency depends on the membrane’s selective affinity towards one of the components of the mixture. The vacuum is generated on the permeate side, whereas the feed side will be at the atmospheric pressure; this leads to the generation of the pressure difference between the two sides, which acts as a driving force for the separation process.

Research in the pervaporation area is mainly directed towards designing an efficient membrane material. The materials must encompass properties such as film-forming ability, high selectivity, higher permeation flux, good mechanical strength, and good shelf life. Research in this direction has shown that hydrophilic materials are always suitable for the dehydration of alcohols, and many such membrane materials have been studied for dehydration of alcohols [10,11,12,13].

Among all the hydrophilic materials, poly(vinyl alcohol) (PVA) is considered to be promising because of its cost-effectiveness, hydrophilicity, and excellent membrane-forming properties [14,15,16,17,18]. However, the PVA membrane suffers from excessive swelling, which leads to poor pervaporation efficiency; to overcome this, many modification techniques such as grafting, crosslinking, blending, as well as the addition of nanomaterials are employed to strengthen the membrane and solve the problem of excessive swelling [19,20].

In recent advances, Hanshuo et al. fabricated PVA/SO_4_^2−^-anodic Al_2_O_3_ membranes by dip coating method. The dual functional flat composite membranes had shown enhanced PV performance [21]. Chaudhari et al. prepared poly(vinyl alcohol)–tetraethyleorthosilicate membrane and modified it through layer by layer deposition of poly(vinyl amine)/silicotungstic acid polyelectrolyte. In terms of the membrane’s layer by layer modification, the authors achieved a higher flux [22]. In order to enhance the PV properties of PVA, Thorat et al. prepared PVA/ionic liquid mixed matrix membranes by solvent evaporation method using four ionic liquids: 1-n-butyl-3-methylimidazolium chloride (BMIMCl), 1-hexyl-3-methylimidazolium chloride (HMIMCl), 1-octyl-3-methylimidazolium chloride (OMIMCl), and 1-hexyl-3-methylimidazolium tetrafluoroborate (HMIMBF4). The membrane performance was based on the number of alkyl groups. It was observed that the higher alkyl groups of cation were responsible for high selectivity towards the water, whereas lower alkyl groups were responsible for high flux [23].

Nanomaterials possess incredible properties such as high thermal stability, high surface area, good optical activity, and conductive properties, which can be utilized to enhance the membrane performance by their incorporation into the membrane matrix [24,25,26]. Amongst all the nanomaterials, graphene is a very interesting nanomaterial as it possesses a two-dimensional layered structure (honeycomb-like structure), which makes it thermally and mechanically highly stable as well as providing it a high surface area. The layered arrangement and small interlayer space make the graphene highly selective and an excellent additive. Moreover, its adhesive nature with silane groups makes it more effective and stable in the membranes [27]. Liange et al. fabricated graphene oxide/polyacrylonitrile composite membranes for pervaporation applications and revealed that graphene oxide-based films show preferential water transport [28]. Dharupaneedi et al. prepared chitosan nanocomposite membranes with embedded functionalized graphene sheets and subjected them to isopropanol and ethanol dehydration via pervaporation. In the study, they also revealed that functionalized graphene sheets enhanced the water permeation and decreased the alcohol permeation through the membrane [29]. These studies created curiosity in us to understand the effect of graphene more systematically.

In this research work, we attempted to study the effect of graphene on the PV efficiency by loading the graphene into the benzaldehyde 2 sulphonic sodium salt acid (B2SA)-grafted PVA–tetraethylorthosilicate (TEOS) hybrid membranes. The morphological and chemical properties of the developed membranes were analyzed by means of FTIR, wide-angle X-ray diffraction (WAXD), thermogravimetric analysis (TGA), SEM, mechanical analysis, and contact angle analysis. Then, the effect of graphene loading on the PV separation of azeotropic aqueous bioethanol was broadly studied.

## 2. Materials and Methods

### 2.1. Materials

Graphene was procured from United Nanotech Innovations Pvt. Ltd., Banglore, India. Tetraethylorthosilicate (TEOS) was procured from E. Merck Ltd., Mumbai, India. Benzaldehyde 2 sulphonic sodium salt acid (B2SA) and poly(vinyl alcohol) were purchased from spectrum reagents and chemicals Pvt. Ltd., Cochin, India. Chemicals purchased were used in the study without further purification as they were reagent-grade chemicals.

### 2.2. Membrane Preparation

**Figure 1:**
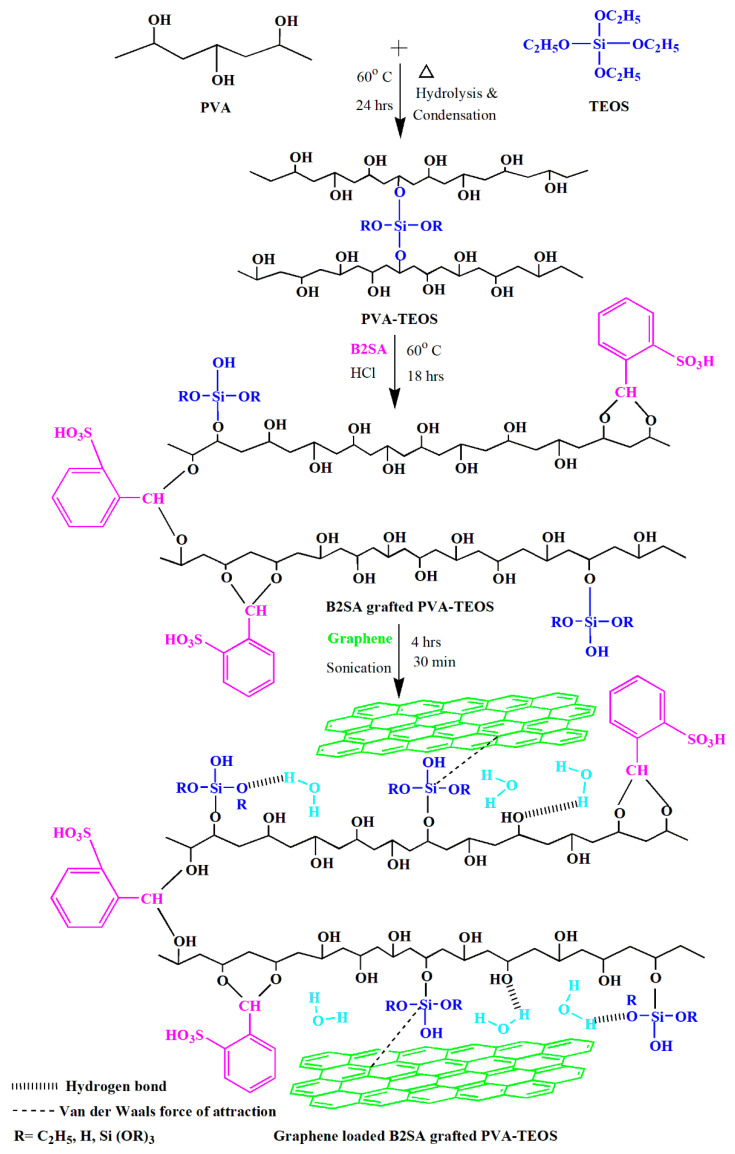
Scheme for the fabrication of graphene-loaded sulfonated poly(vinyl alcohol) (PVA)–tetraethylorthosilicate (TEOS) hybrid membrane.

The PVA solution (4 mass%) was made by dissolving the PVA in double-distilled water and kept for constant stirring for half a day. The solution was then filtered to remove the undissolved particles. To the above solution, we mixed in a known quantity of tetraethylorthosilicate (6 g), and the mixture was stirred for 1 day at 60 °C. The resultant homogenous solution was then casted on a clean and smooth glass plate by means of a casting knife in pollution-free atmosphere. The membrane was allowed to dry properly at room temperature. A fully dried membrane was then peeled off and named as M. B2SA-grafted hybrid PVA membrane was prepared by mixing a known quantity of B2SA (7.5 mass%) to the above-prepared solution and stirred for 1 day at 60 °C. The remaining procedure followed was similar to the procedure followed for the hybrid PVA membrane preparation and subsequently the prepared membrane was named as M-B2SA [19]. With the intention of studying the effect of graphene on pervaporation performance of sulfonated PVA–TEOS hybrid membranes, known amounts (0.5%, 1%, 1.5%, and 2%) of different mass% of graphene were added with respect to PVA and stirred for 4 h, being kept in a sonicator for 30 min to obtain a uniform suspension. Further, the suspension was spread on a glass plate and subjected to drying for 3 days. Then, the membranes were carefully peeled off and were named as M-0.5 G, M-1 G, M-1.5 G, and M-2 G, respectively. Upon further enhancement in the content of graphene, the membrane peeling became difficult because of agglomerated graphene. Therefore, we stopped the membrane development at 2 mass% graphene. The thickness of the developed membranes was assessed at various positions using a thickness gauge (Peacock dial thickness gauge). The uniform thickness 50 ± 2 μm was obtained for the fabricated membranes. The membrane preparation scheme for the graphene loaded sulfonated PVA–TEOS hybrid membrane is illustrated in Figure 1.

### 2.3. Fourier Transform Infrared (FTIR) Spectroscopy

Spectrum two FTIR with Diamond ATR (PerkinElmer Singapore Pte. Ltd., 28, Ayer Rajah Crescent, no. 08-01, Singapore 139959) was used to study the interactions between the chemicals used in the developed membranes. FTIR analysis was recorded in the range of 500 to 4000 cm^−1^. KBr method was employed for the analysis [30].

### 2.4. Wide-Angle X-ray Diffraction (WAXD)

Philips Analytical X-ray Diffractometer (Rigaku SmartLab SE, Tokoyo, Japan) was used to analyze the solid-state morphology of the developed membranes. Membrane samples were scanned in the range 5° to 50° for the angle 2*θ* at the rate of 8°/min.

### 2.5. Differential Scanning Calorimetry (DSC)

Differential scanning calorimeter (DSC Q20, TA Instruments, Waters LLC., New Castle, DE, USA) was used to study the crystallinity of fabricated membranes. Weight of samples ranging from 9 to 10 mg were subjected to heating from atmospheric temperature to 700 °C under nitrogen atmosphere at a heating rate of 10 °C per minute.

### 2.6. Thermogravimetric Analysis (TGA)

A thermogravimetric analyzer named SDT Q600 (TA Instruments, Waters LLC., New Castle, DE, USA) was used to study the thermal analysis of the fabricated membranes under the nitrogen atmosphere. The heating rate was maintained at 10 °C/min.

### 2.7. Scanning Electron Microscopy (SEM) and Energy Dispersive X-ray (EDX) Analysis

The surface morphology and elemental composition of the graphene was analyzed using a scanning electron microscope (SEM) and energy dispersive X-ray (EDX) analysis named JEOL-JSM-IT500, (Tokyo, Japan). Further, membranes were examined using a scanning electron microscope (SEM), and all the membranes were dried properly prior to the analysis and were sufficiently covered with a sputtered gold layer of 400 Å.

### 2.8. Mechanical Properties

The mechanical strength and elastic nature of the developed membranes were analyzed using the Universal Testing Machine (DAK system Inc., Maharastra, India). The analysis was performed 3 times, and the mean value was considered.

### 2.9. Swelling Measurement

Membrane sorption measurements were conducted at room temperature by considering the azeotropic mixture of water and bioethanol. Initially, the developed membranes were vacuum dried and weighed. Further, these membranes were then dipped in the azeotropic composition of bioethanol for 1 day in a closed bottle for equilibrium establishment. After this, the swollen membranes were carefully blotted and weighed. The percentage membrane sorption was calculated using the expression [31,32,33]:

(1) $$DS(\%)=\left(\frac{W_{s}-W_{d}}{W_{d}}\right)\times 100$$

where *W_d_* and *W_s_* are the masses of the dry and swollen membranes, respectively.

### 2.10. Contact Angle Meter

To study the surface properties of the developed membranes at 30 °C, we measured the contact angle by the sessile drop method by means of a contact angle meter named Kyowa Interface measurement and analysis (Japan).

### 2.11. Pervaporation Experiments

**Figure 2:**
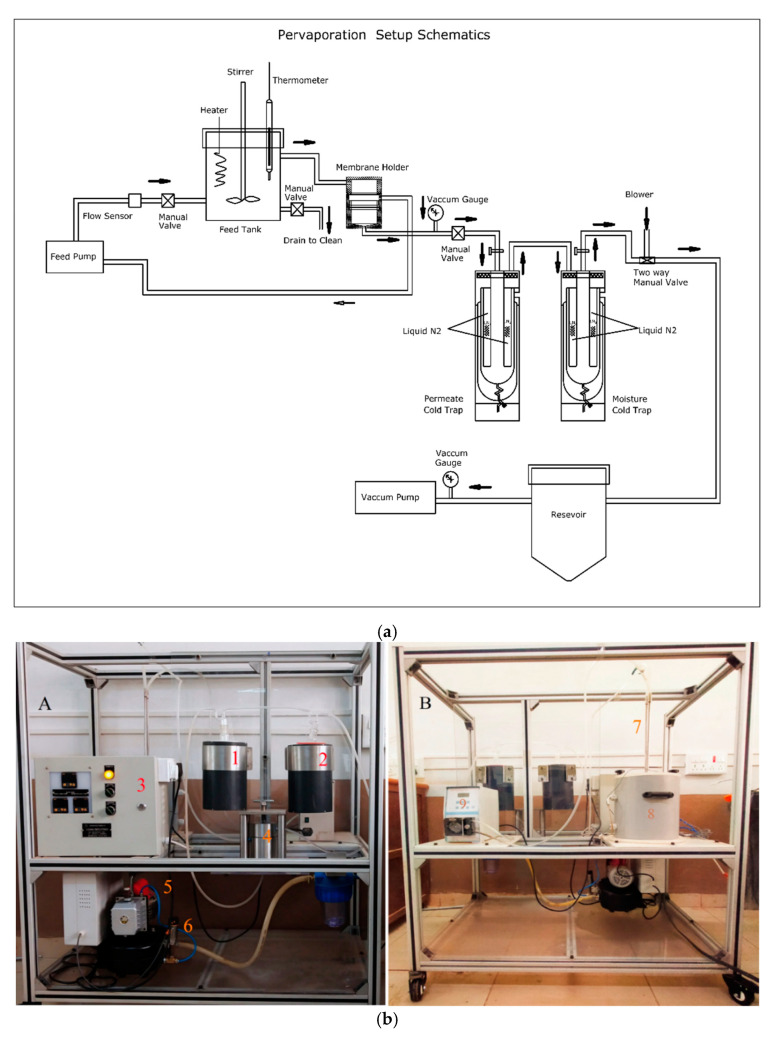
(**a**) Schematic representation of the pervaporation unit. (**b**) Photographic image of the pervaporation apparatus: (**A**) front view: (1) permeate cold trap; (2) moisture cold trap; (3) control panel; (4) pervaporation cell; (5) vacuum pump; (6) vacuum control sensor. (**B**) Back view: (7) inlet and outlet of the feed tank; (8) feed tank; (9) circulation pump.

A custom-made apparatus was used to carry out the pervaporation experiments. The schematic design of the pervaporation apparatus is illustrated in Figure 2a.

The membrane with the surface area 15 cm^2^ was sandwiched between permeate and the feed section. The feed section was equipped with a stirrer aided by a DC motor to maintain the uniform temperature and flow of the liquid mixture. The photographic image of the designed PV unit [34] is illustrated in Figure 2b. The membrane under the test was allowed to equilibrate with the feed mixture for about 1 h in the feed section at room temperature. Once the membrane attained the equilibrium in the PV apparatus, a vacuum of 31.325 kPa was induced. On the permeate section, vapors were condensed in the cold trap and collected in the form of liquid at the uniform interval of 1 h time periods. After that, the weight of the permeate was measured using a digital microbalance. KAFI smart Karl Fischer Titrator was used to analyze the composition of the permeate liquid in terms of percentage. The experiment was repeated 3 times, and the average results were considered. Then, the PV performance of the membranes was analyzed by measuring the separation selectivity (*α_sep_*), total permeation flux (*J*), and pervaporation separation index (*PSI*) using the following expressions [35,36]:

(2) $$J=\frac{W}{A.t}$$

(3) $$\alpha_{sep}=\frac{P_{w}/P_{ET}}{F_{w}/F_{ET}}$$

(4) $$PSI=J(\alpha_{sep}-1)$$

where *A* is the membrane area (m^2^); *W* is the mass of permeate (kg); *t* is the permeation time (h); *P_ET_* and *P_w_* are the mass percent of ethanol and water in the permeate, respectively; and *F_ET_* and *F_w_* are the respective mass percent of ethanol and water in the feed.

The permeance (*Pi/l*) was measured by using the following expression for all the membranes to obtain a clear idea about the fundamental properties of the membranes [37,38]:

(5) $$\frac{P_{i}}{l}=\frac{D_{i}K_{i}}{l}=\frac{j_{i}}{P_{i}^{f}-P_{i}^{p}}$$

In this expression, *K_i_* and *D_i_* are the sorption and diffusion coefficient of the *i*th constituent, respectively; *P_i_* is the permeability of the *i*th constituent, $P_{i}^{P}$ and $P_{i}^{f}$ are the vapor pressures of the *i*th constituent in permeate and feed, respectively; *j_i_* is the molar flux of *i*th component; and *l* is the membrane thickness.

## 3. Results

### 3.1. Membrane Characterization

#### 3.1.1. FTIR Studies

**Figure 3:**
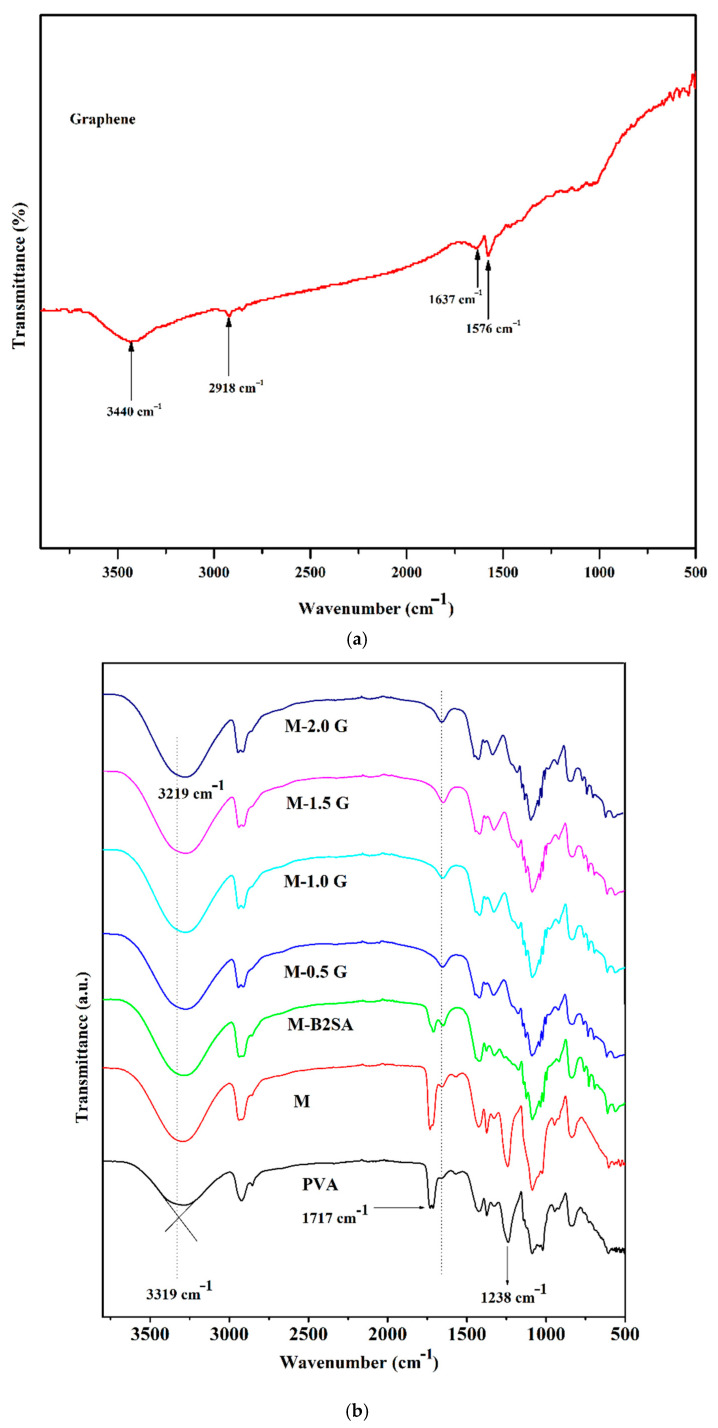
(**a**) FTIR spectra of plane graphene. (**b**) FTIR spectra of PVA; PVA–TEOS (M); sulfonated PVA–TEOS (M-B2SA); and graphene-loaded, sulfonated PVA–TEOS hybrid membranes: (M-0.5 G) 0.5 mass%; (M-1 G) 1 mass%; (M-1.5 G) 1.5 mass%; (M-2 G) 2 mass% of graphene.

The FTIR spectra of pure graphene is illustrated in Figure 3a. In the FTIR of pure graphene, the peak appearing at 3440 cm^−1^ was due to the presence of atmospheric moisture. The peak visible at 1637 cm^−1^ was due to the skeletal vibrations of the graphene backbone chain. All these characteristic peaks confirmed the structure of graphene [26].

The FTIR spectra of PVA; PVA–TEOS; B2SA-grafted PVA–TEOS; and graphene-loaded, B2SA-grafted PVA–TEOS hybrid membranes are illustrated in Figure 3b. In the FTIR of PVA, a broad peak appearing around 3319 cm^−1^ corresponded to the O–H stretching vibrations. A small peak appearing at 840 cm^−1^ was due to the C–C stretching vibrations. Multiple bands appearing in the range of 1000 cm^−1^ and 1100 cm^−1^ corresponded to C–O of PVA and Si–O–C linkage formed because of crosslinking between PVA and TEOS. Further, the increase in the intensity observed in the multiple bands present between 1000 cm^−1^ and 1100 cm^−1^ confirmed the crosslinking reaction between the PVA and TEOS [39].

A broad peak appearing at around 3319 cm^−1^ corresponded to the O–H group stretching vibrations. A small peak at around 730 cm^−1^ appeared in the sulfonated PVA–TEOS and graphene-loaded sulfonated PVA–TEOS membranes, which was due to the presence of the B2SA content as the peak indicated aromatic alkene out of plane bending. Multiple peaks appearing between the ranges 1000 to 1200 cm^−1^ corresponded to C–O groups of acetal linkage between the –OH group of PVA and the –CHO group of B2SA in hybrid PVA membranes [40]. The peak around 1710 cm^−1^ corresponding to –CHO group of B2SA was missing in the spectra, which conformed to the reaction between B2SA and PVA. Moreover, the peak that appeared due to the acetate groups in PVA almost disappeared in M-B2SA and graphene-incorporated membranes, suggesting the acid hydrolysis reaction [19]. Further, the broad intensity peak appearing at 3318 cm^−1^ was enhanced systematically, which was due to the presence O–H groups of water molecules present in the channels created due to the adhesion (Van der Waals force of attraction) between the graphene and the silane group of TEOS. This peak was systematically augmented as the content of graphene enhanced in the hybrid PVA matrix [41]. The intensity of the peak observed at around 1640 cm^−1^ was enhanced from sulfonated PVA–TEOS hybrid membrane to graphene-loaded, sulfonated PVA–TEOS. This peak represented the O–H bending vibrations of the bound water molecules. Further, the broad intensity peak appearing at 3318 cm^−1^ was enhanced along with the slight shift towards the lower wavenumber, which was due to the hydrogen bonding and rearrangement of polymer chains because of the graphene incorporation in the membrane matrix. All this spectral evidence supports the crosslinking between PVA–TEOS, B2SA in the fabricated hybrid membranes, and graphene presence in the membrane matrix.

#### 3.1.2. Wide-Angle X-ray Diffraction Studies (WAXD)

**Figure 4:**
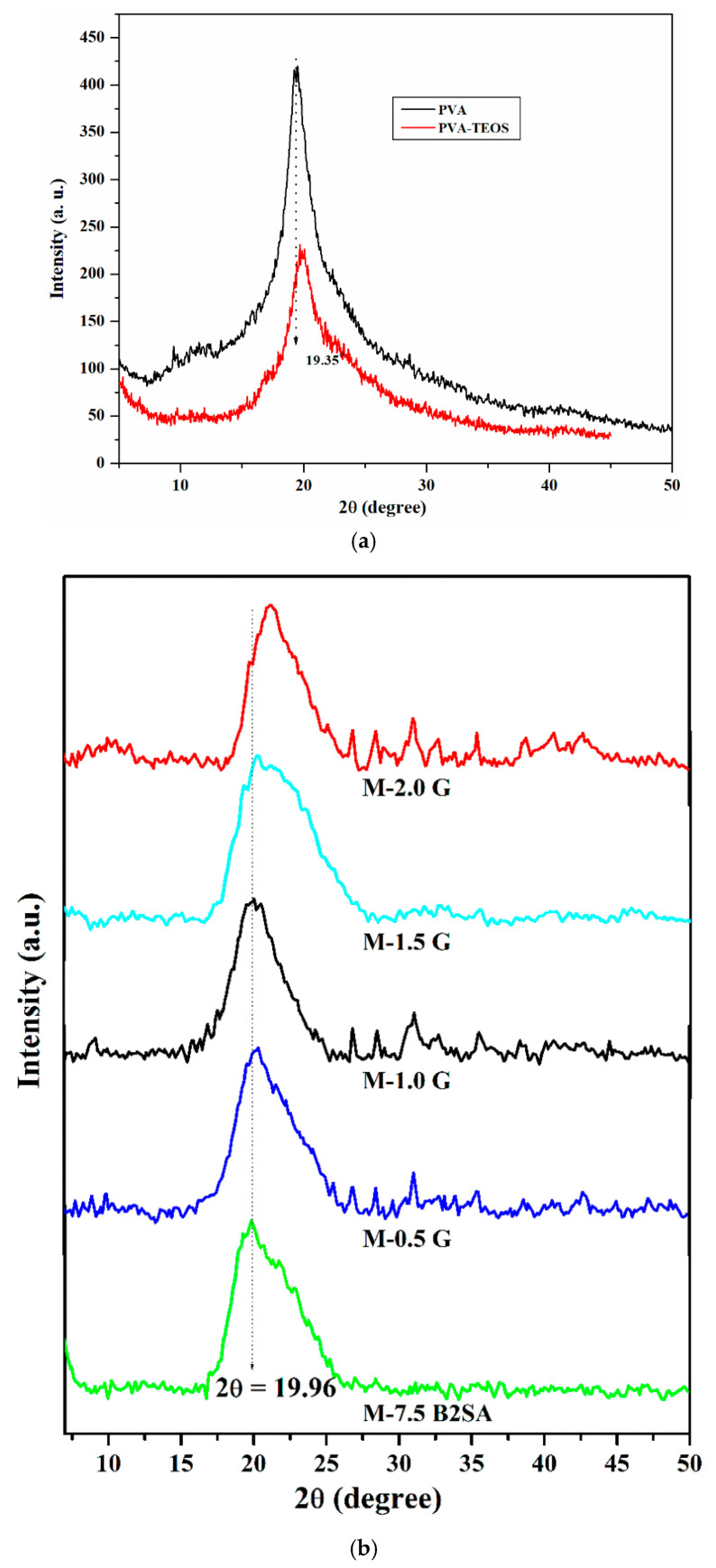
(**a**) Wide-angle X-ray diffraction patterns of plane PVA and PVA–TEOS membranes. (**b**) Wide-angle X-ray diffraction patterns of sulfonated PVA–TEOS (M-B2SA) and graphene-loaded sulfonated PVA–TEOS hybrid membranes: (M-0.5 G) 0.5 mass%; (M-1 G) 1 mass%; (M-1.5 G) 1.5 mass%; (M-2 G) 2 mass% of graphene.

It is necessary to study the WAXD patterns of the PVA and PVA–TEOS membranes to initially understand the effect of graphene on the hybrid membranes. Therefore, the developed membranes were subjected to WAXD analysis, and the resulting patterns are illustrated in Figure 4a. From the WAXD pattern observed for PVA, we found a peak at 2*θ* = 19.35° with the d-spacing value of 4.58 Å. This peak corresponded to the degree of crystallinity of PVA [42]. Further, in the case of TEOS-crosslinked PVA membranes, the peak was shifted to a higher angle of 19.72° along with the decrement in the d-spacing value (4.5 Å) and intensity. This confirmed the reaction between –OH groups of PVA and silanol groups of TEOS. The lowered d-spacing value indicated the structural rearrangement observed in the polymer matrix due to the crosslinking reaction and was evidence for the enhanced compact structure.

In order to analyze the effect of graphene on the solid-state morphology of the plane PVA and sulfonated PVA–TEOS hybrid membranes, we subjected the developed membranes to WAXD analysis, and the resulting patterns are illustrated in Figure 4b; here, we compare the WAXD patterns of TEOS-crosslinked PVA membranes with the B2SA-grafted PVA–TEOS hybrid membrane, finding that the intensity of the peak further decreased slightly but the d-spacing value remained constant (4.50 Å). Further, for graphene-loaded, B2SA-grafted PVA–TEOS hybrid membranes, the peak was shifted to a higher 2*θ* angle. This resulted in the lower d-spacing values of the graphene-loaded, B2SA-grafted PVA–TEOS hybrid membranes, with M-2 G showing the lowest d-spacing value of 4.36 Å. This was due to the lowered inter-chain distance between the crosslinked polymer chain structure due to the interaction between graphene and the silane groups of TEOS. This makes the graphene-loaded, B2SA-grafted PVA–TEOS hybrid membrane more selective compared to the B2SA-grafted PVA–TEOS hybrid membrane.

#### 3.1.3. Differential Scanning Calorimetry (DSC)

**Figure 5:**
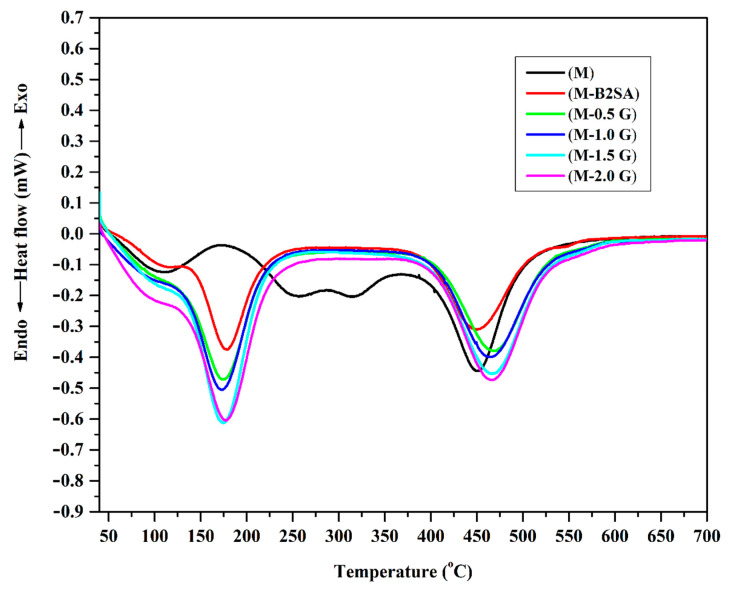
Differential scanning calorimetry (DSC) thermograms of PVA–TEOS (M); sulfonated PVA–TEOS (M-B2SA); and graphene-loaded, sulfonated PVA–TEOS hybrid membranes: (M-0.5 G) 0.5 mass%; (M-1 G) 1 mass%; (M-1.5 G) 1.5 mass%; (M-2 G) 2 mass% of graphene.

From the obtained DSC thermogram of the fabricated membranes (Figure 5), we noticed that for the hybrid PVA membrane, glass transition temperature (T*_g_*) was observed around 106 °C, which was higher compared to the T*_g_* of plane PVA membranes found in the literature [43]. This confirmed the successful crosslinking and sulfonation of the PVA. Further, an endothermic peak was observed at 178 °C for the hybrid PVA membrane, which was due to the release of bound water molecules. Bound water molecules will be eliminated at a higher temperature because the bound water molecules are chemically linked with –OH groups of PVA. Observing carefully, we were able to see that as the graphene addition took place, the peak observed at 178 °C for hybrid PVA membrane slightly shifted to the lower temperature, and the area under the curve enhanced substantially, which indicated the higher water-holding capacity of the membranes. Out of the given membranes, the membranes containing graphene exhibited the highest area under the curve, which indicated that the membrane possessed the highest water-holding capacity out of all the synthesized membranes. This enhancement in the water-holding capacity was due to the channels created in the membrane matrix due to the interactions between graphene and silane groups of TEOS, which can accommodate the monolayer of water. The decomposition peak for the hybrid PVA membrane was observed at 448 °C, whereas the graphene-loaded hybrid PVA membranes were 466 °C. This was evident as the incorporation of graphene in the membrane matrix will make the membranes more thermally stable.

#### 3.1.4. Thermogravimetric Analysis (TGA)

**Figure 6:**
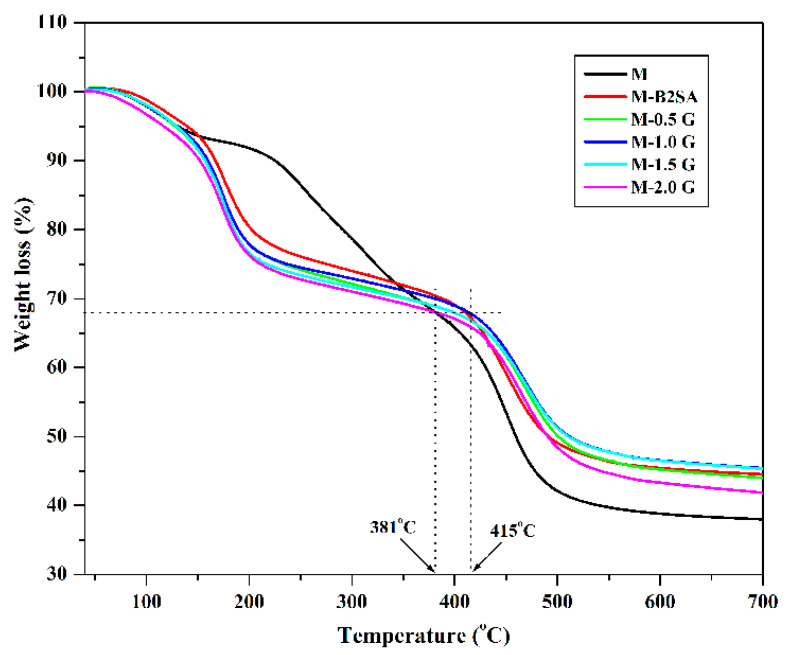
Thermogravimetric analysis patterns of PVA–TEOS (M); sulfonated PVA–TEOS (M-B2SA); and graphene-loaded, sulfonated PVA–TEOS hybrid membranes: (M-0.5 G) 0.5 mass%; (M-1 G) 1 mass%; (M-1.5 G) 1.5 mass%; (M-2 G) 2 mass% of graphene.

The thermograms of the fabricated membranes can inform the thermal stability of the membranes. The thermograms obtained for the fabricated membranes are depicted in Figure 6. The thermograms indicated that the nonoxidative degradation of the fabricated membranes occurred in three phases. The first stage decomposition occurred between ambient temperature to 190 °C is due to the desorption of moisture. The second stage decomposition occurred between the temperature range of 190–420 °C. This decomposition was due to the loss of functional groups such as hydroxyl and sulfonated phenyl groups. Finally, the third stage decomposition was observed in the temperature range of 420–520 °C, which was due to the breakdown of the polymeric chains. If we consider 32% weight loss as a reference point, then for the PVA–TEOS membrane, the temperature observed was 381 °C, whereas for membrane M-2 G, the temperature was 415 °C. This indicates the higher thermal stability of the graphene-loaded, B2SA-grafted PVA–TEOS hybrid membranes compared to the PVA–TEOS and B2SA-grafted PVA–TEOS hybrid membranes. This higher stability was due to the presence of graphene, which formed an interface that restricted the thermal motion of the polymer chains. Fabricated membranes exhibited good stability for pervaporation experiments as these membranes were thermally stable up to 100 °C.

#### 3.1.5. Scanning Electron Microscopy (SEM)

**Figure 7:**
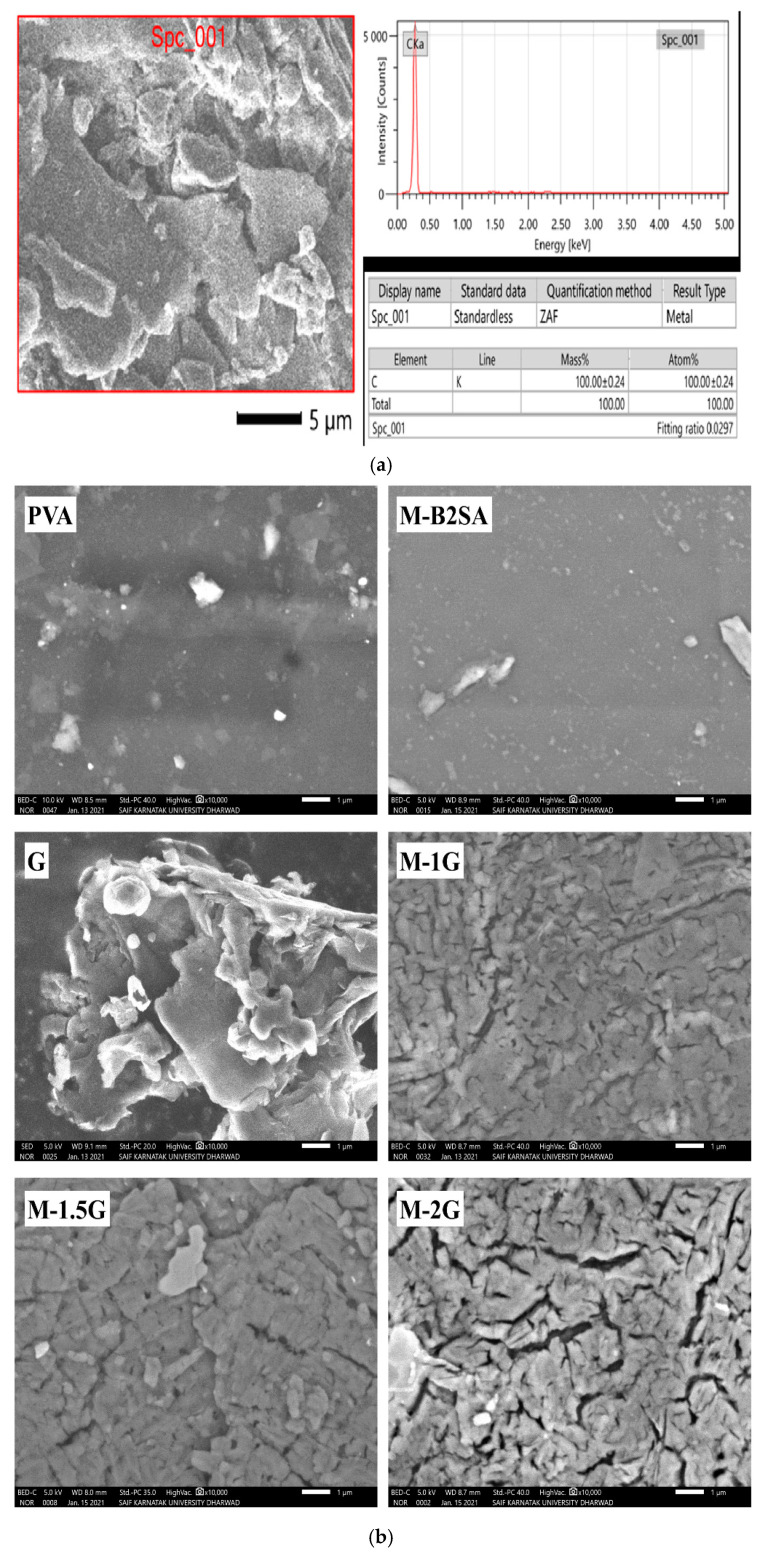
(**a**) SEM and energy dispersive X-ray (EDX) specta of graphene. (**b**) SEM micrographs of PVA; graphene; sulfonated PVA–TEOS (M-B2SA); and graphene-loaded, sulfonated PVA–TEOS hybrid membranes: (M-1 G) 1 mass%; (M-1.5 G) 1.5 mass%; (M-2 G) 2 mass% of graphene.

The SEM micrograph of graphene is depicted in Figure 7a at 5.0 kx magnifications at a voltage of about 20 kV. This micrograph demonstrated that the graphene showed the characteristic wrinkled and scrolled intrinsic flat sheet-like morphology. The grapheme was further analyzed for EDX to obtain the elemental composition of graphene. The EDX profile of graphene showed the signals corresponding to carbon with a mass% of 100. This confirms that graphene contained only carbon atoms without any impurities or residual oxygen atoms.

In order to analyze the uniform dispersion of graphene in the membrane matrix, we carried out SEM analysis of the graphene-loaded, sulfonated PVA–TEOS hybrid membranes, with results being illustrated in Figure 7b. As we carefully analyzed the images, we observed that there were white patches on the graphene-incorporated membranes that were clearly not observed in the PVA and sulfonated PVA–TEOS membranes. These white patches were nothing but graphene layers. Further, as the amount of graphene was enhanced, the brightness of these patches was also increased. This uniform distribution of the graphene may have been due to Van der Waals force of attraction between the graphene and the silane groups of TEOS.

#### 3.1.6. Mechanical Properties

Mechanical properties of the fabricated membranes are important parameters to analyze the stability of the membranes for PV applications. Table 1 provides the information of the elongation at break and the tensile strength values of the fabricated membranes.

From the table, it was clearly seen that the tensile strength gradually enhanced from membrane-sulfonated PVA–TEOS hybrid membrane to M-1.5 G. This was due to the presence of graphene, which will induce strength in the membrane by restricting the movement of the polymer chains near the interface. Further, the tensile strength of the membrane “M-2 G” decreased slightly (21.11 MPa); this may have been due to the formation of graphene aggregations, which consequently initiated the fracture of the membranes.

Reduction in percentage of elongation at break was also observed with enhancement in the content of graphene. This was due to the strong interaction between the polymer matrix and the graphene. These strong interactions led to restricted chain mobility, which further led to a lack of expansiveness of the fabricated membranes. However, the fabricated membranes displayed good mechanical strength and stability for pervaporation.

#### 3.1.7. Contact Angle Analysis

Contact angle analysis is the method used to understand the hydrophilicity of the membranes. Table 2 illustrates the effect of graphene loading on the contact angle values of the fabricated membranes.

From the table, it is clearly seen that the contact angle values systematically decreased with an increase in the concentration of graphene in the membrane matrix. This is due to the arranged graphene layers between the polymer chains, which form nanocapillary channels whose diameter is sufficient to accommodate only the monolayer of water. Hence, the higher the graphene content in the membrane matrix, the lower the contact angle. Moreover, the functional groups such as –OH and –SO_3_H also contributed to the overall hydrophilicity of the membranes. These results were in accordance with the results obtained in sorption studies.

### 3.2. Effects of the Amount of Graphene on Membrane Swelling

Sorption studies or swelling measurements is a method used to study the extent of swelling observed in the fabricated membranes. Table 3 illustrates the extent of swelling observed in the fabricated membranes. From the figure, it is shown that the extent of swelling increased from membrane M to M-2 G, with M-2 G exhibiting the highest percentage of the degree of swelling (12.4%). This is due to the arranged graphene layers between the polymer chains forming nano-capillary channels whose diameter is sufficient to accommodate only the monolayer of water. Therefore, as the graphene content in the membrane increased, a larger amount of water molecules were held by the membranes, leading to the enhanced membrane swelling. This enhanced selectivity towards the water increased the efficiency of PV performance of the membranes by enhancing separation selectivity. These results were also reflected in contact angle analysis.

### 3.3. Effects of the Amount of Graphene on Pervaporation

In pervaporation experiments of these fabricated membranes, the permeation took place through molecular sieving and diffusion mechanisms. The introduction of graphene in the membrane matrix created the number of nanochannels for the permeation process because of the stacked graphene layers in the polymer matrix. Further, as the amount of graphene increased, more such channels were created, which led to higher permeation. The cracks observed on the surface of the graphene-loaded, sulfonated PVA–TEOS membranes in the SEM analysis acted as routes for the permeation of water, as these narrow nanochannels were sufficient enough to accommodate monolayer of water. This led to the higher selectivity of the developed membranes [44]. Pervaporation performance of the membranes at different temperatures is given in Table 4.

As observed from the table, selectivity, permeance, and total permeation flux data of the fabricated graphene-loaded hybrid membranes were augmented as the content of graphene was enhanced in the fabricated membrane. A similar trend of increment was also observed in sorption studies. The enhancement seen in the total permeation flux was linear from sulfonated PVA–TEOS hybrid membrane to M-2 G, with M-2 G showing the 11.66 × 10^−2^ kg/(m^2^ h) total permeation flux at 30 °C. This was due to the arrangement of graphene layers between the polymer chains, which made the membrane matrix denser and reduced the inter-chain space, as observed in the WAXD and DSC analyses. The inter-chain distance observed was sufficient enough to accommodate the monolayers of water because of the nanochannels created by the graphene. The presence of graphene in the membrane matrix enhanced the diffusion selectivity and sorption towards water molecules, which enhanced the overall permeation flux. Moreover, graphene incorporated in the membrane was not only responsible for the enhancement in water affinity but also responsible for declined crystalline regions of the membrane, which enhanced the diffusion mechanism through the membrane.

**Figure 8:**
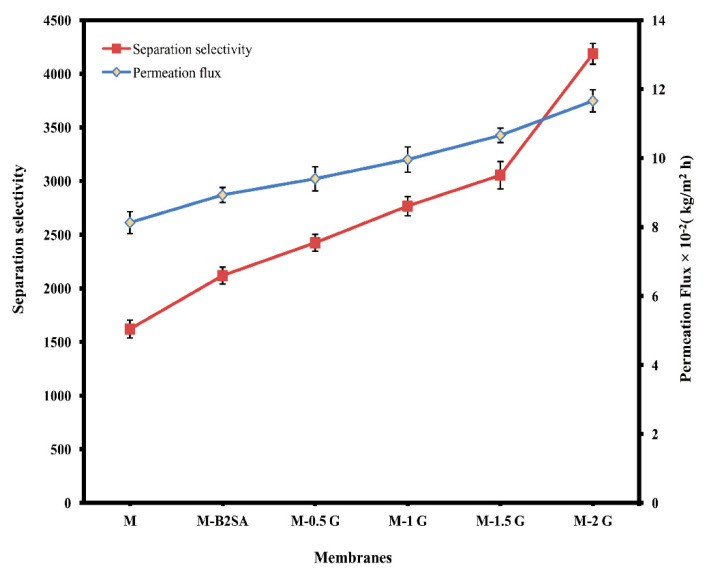
Deviation in total permeation flux and separation selectivity of water and ethanol with different mass% of G at 30 °C.

The separation selectivity of the membrane was largely dependent on the size of the permeating molecules and the free volume of the polymer membrane matrix. From the data, we noted that separation selectivity was consistently enhanced upon the increase in the content of graphene. This was due to the selective nature of graphene towards the water. The arranged graphene layers between the polymer chains formed nanocapillary channels whose diameter would be sufficient to accommodate only the monolayer of water. Moreover, decreased d-spacing value resulted in selective diffusion in which bioethanol, being a larger sized molecule (molecular diameter 0.44 nm) compared to the water (molecular diameter of water is 0.28 nm), was held back [29]. Enhancement in both total permeation flux and separation selectivity was not commonly observed because of the trade-off phenomenon. However, in this study, the separation selectivity and permeation flux was enhanced systematically from the sulfonated PVA–TEOS hybrid membrane to M-2 G. The variation of permeation flux and separation selectivity with the graphene content is shown in Figure 8.

**Figure 9:**
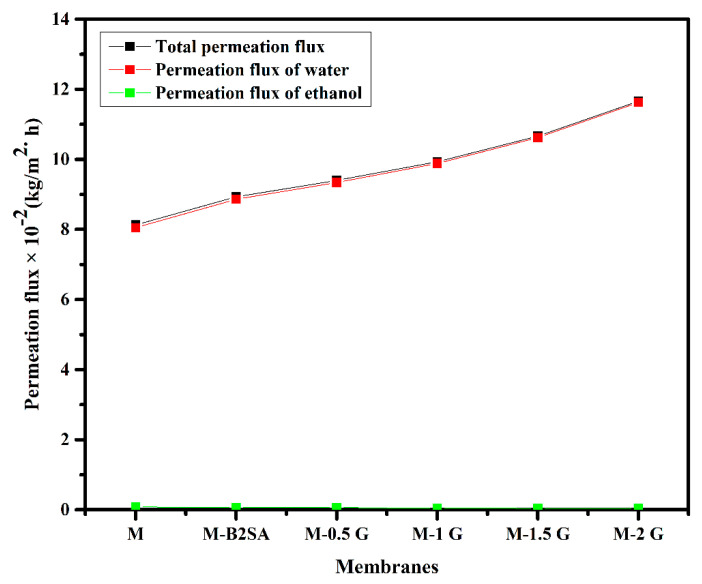
Deviation in total permeation flux and permeation fluxes of water and ethanol at 30 °C.

Plots of the individual permeation fluxes of water and bioethanol and the total permeation flux considered as a function of graphene content are represented in Figure 9.

From the plots, it can be seen that the curves representing total permeation flux and permeation flux of water almost overlapped each other. In comparison, the permeation flux of bioethanol was almost negligible. This was evident in the selective nature of the fabricated membranes towards the water. The permeation of individual component of water and ethanol at various temperatures is illustrated in Table 5.

### 3.4. Effect of Graphene on the Pervaporation Separation Index (PSI)

**Figure 10:**
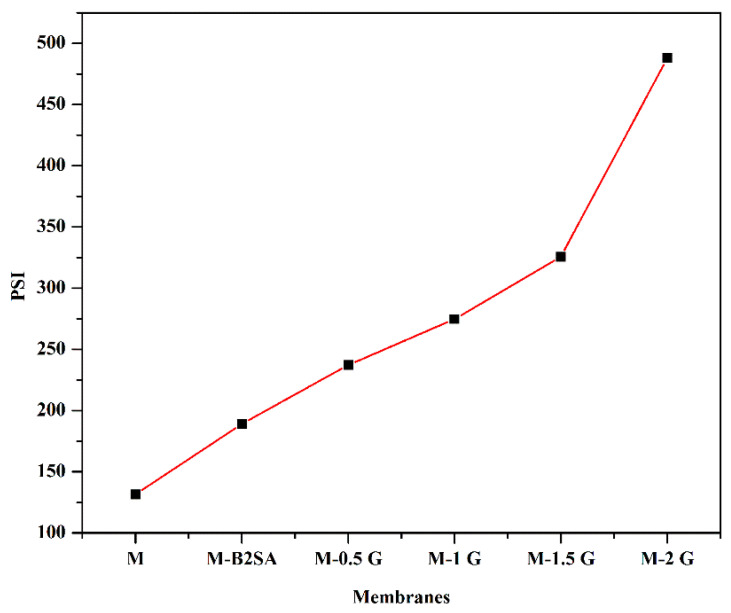
Effect of graphene content on pervaporation separation index (PSI) at 30 °C.

With the intention to understand the overall performance of the membrane, we calculated the pervaporation separation index (*PSI*) for the fabricated membranes at 30 °C. It is one of the important factors to measure membrane efficiency. The graph of *PSI* considered as a function of graphene content is represented in Figure 10. From the plot, one can notice that there was a systematic increment in the values as the graphene content was enhanced in the membrane. This was expected as the separation selectivity enhanced simultaneously as graphene content was enhanced in the membrane. M-2 G membrane demonstrated the maximum value (416) out of the fabricated membranes, which showed that it exhibited good efficiency for pervaporation separation of water from bioethanol.

### 3.5. Effect of Temperature on Membrane Performance

From Table 4, we can see that total permeation flux was enhanced with increment in temperature. This may have occurred due to the following reasons. Firstly, a pressure difference may have been created between the feed and the permeate side as a result of the increment in the temperature, leading to the molecular diffusion in the membrane. Secondly, the increased temperature may have not only led to an increment in free volume, but it also could have supported thermal motion in polymer chains. Nevertheless, the second reason can be ruled out as the highest temperature maintained in the experiments was 50 °C. Therefore, we can come to the conclusion that the temperature acted as a driving force for the diffusion of molecules through the membrane, which was incremental in the permeation flux and hampered the separation selectivity.

## 4. Conclusions

In this research analysis, graphene-loaded, sulfonated PVA–TEOS hybrid membranes were developed by a solution casting method. These membranes were characterized by means of various characterizing techniques. In FTIR, the broad intensity peak appearing at 3318 cm^−1^ was enhanced systematically, which was due to the presence of O–H groups of water molecules present in the channel created due to the adhesion (Van der Waals force of attraction) between the graphene and the silane group of TEOS. From the WAXD patterns, it was revealed that as the graphene was incorporated in the B2SA-grafted PVA–TEOS hybrid membrane, the peak, which was at 2*θ* = 19.65°, was shifted to a higher 2*θ* angle of 20.32°, along with the drastic increment in the intensity of the peak. A higher 2*θ* angle also resulted in the lower d-spacing value of 4.36 Å, which resulted in enhanced selectivity of the membrane. TGA showed the higher thermal stability of the membrane after the incorporation of graphene into the matrix. DSC revealed that the graphene-incorporated membranes exhibited a higher area under the curve, which indicated that the membranes with graphene possessed a higher water-holding capacity. These results were also reflected in sorption studies and contact angle measurements, as these characterizations also revealed that the membrane M-2 G exhibited higher water-holding capacity. Enhancement in both total permeation flux and separation selectivity is not commonly observed because of the trade-off phenomenon. However, in this study, both the separation selectivity and the total permeation flux were enhanced simultaneously.

## Figures and Tables

**Table 1:** Mechanical properties of the graphene-loaded hybrid PVA membranes.

Membrane	Tensile Strength (MPa)	Elongation at Break (%)
M	15.07 ± 1.26	56 ± 3.26
M-B2SA	18.56 ± 2.19	47 ± 2.21
M-0.5 G	19.87 ± 2.80	45 ± 3.02
M-1.0 G	21.09 ± 2.98	38 ± 3.25
M-1.5 G	22.23 ± 2.00	37 ± 3.23
M-2.0 G	21.11 ± 2.03	35 ± 2.71

**Table 2:** Contact angles of the developed membranes.

Membrane	Contact Angle (°)
M	60 ± 2.1
M-B2SA	57 ± 1.5
M-0.5 G	54 ± 3.3
M-1.0 G	51 ± 3.5
M-1.5 G	48 ± 1.2
M-2.0 G	44 ± 2.9

**Table 3:** The percentage degree of swelling of the developed membranes.

Membrane	Percentage Degree of Swelling
M	8.1 ± 1.2
M-B2SA	9.4 ± 1.8
M-0.5 G	9.9 ± 2.0
M-1.0 G	10.6 ± 2.4
M-1.5 G	11.8 ± 2.9
M-2.0 G	12.4 ± 3.1

**Table 4:** Permeation flux, separation selectivity, and permeance data of the developed membranes at various temperatures ^a^.

Membrane	Temperature (°C)	*J* (kg/m^2^h)	*α_sep_*	*P_i_*/*l* (GPU)
M	30	0.0813	1620	1626
	40	0.0900	1476	1800
	50	0.1000	1413	2000
M-B2SA	30	0.0893	2119	1786
	40	0.0993	1836	1986
	50	0.1113	1728	2226
M-0.5 G	30	0.0940	2425	1880
	40	0.1033	2178	2066
	50	0.1206	1976	2412
M-1 G	30	0.0993	2767	1986
	40	0.1120	2425	2240
	50	0.1320	2158	2640
M-1.5 G	30	0.1066	3055	2132
	40	0.1146	2673	2292
	50	0.1406	2262	2812
M-2.0 G	30	0.1166	4187	2332
	40	0.1233	3405	2466
	50	0.1533	2703	3066

^a^ GPU = gas permeation unit = 10^−6^ cc (Standard Temperature and Pressure)/cm^2^/s/cm Hg.

**Table 5:** Permeation flux of water and permeation flux of ethanol for all the membranes for different temperatures.

Temp. °C		*J* × 10^2^ kg/(m^2^ h) for Water					*J* × 10^2^ kg/(m^2^ h) for Ethanol					
	M	M-B2SA	M-0.5 G	M-1 G	M-1.5 G	M-2 G	M	M-B2SA	M-0.5 G	M-1 G	M-1.5 G	M-2 G
30	8.0113	8.8299	9.3078	9.8446	10.5832	11.5935	0.1186	0.1000	0.0921	0.0854	0.0767	0.0665
40	8.8560	9.8019	10.2174	11.0902	11.3580	12.2437	0.1440	0.1281	0.1126	0.1098	0.1020	0.0863
50	9.8330	10.9775	11.9153	13.0548	13.9124	15.1951	0.1670	0.1525	0.1447	0.1452	0.1476	0.1349

## Abbreviations

Nomenclature	
*M_w_*	molecular weight
*A*	effective membrane area (m^2^)
*DS*	degree of swelling (%)
ET	ethanol
*J*	Total permeation flux (kg/m^2^h)
*J_o_*	pre-exponential factor for permeation
*PSI*	pervaporation separation index
*P* and *F*	mass percent of permeate and feed
*T*	permeation time (h)
*T*	temperature (K)
*W*	mass of permeate (kg)
*W_s_* and *W_d_*	mass of the swollen and dry membranes
Greek letters	
Δ	membrane thickness
*α_sep_*	separation factor
